# Accounting for imperfect detection when estimating species‐area relationships and beta‐diversity

**DOI:** 10.1002/ece3.70017

**Published:** 2024-07-10

**Authors:** Ciar D. Noble, Carlos A. Peres, James J. Gilroy

**Affiliations:** ^1^ School of Environmental Sciences University of East Anglia Norwich, Norfolk UK; ^2^ Instituto Juruá Manaus Brazil

**Keywords:** Chao estimator, community simulation, habitat fragmentation, iNEXT.3D, multi‐species occupancy model, species richness

## Abstract

Ecologists have historically quantified fundamental biodiversity patterns, including species‐area relationships (SARs) and beta diversity, using observed species counts. However, imperfect detection may often bias derived community metrics and subsequent community models. Although several statistical methods claim to correct for imperfect detection, their performance in species‐area and β‐diversity research remains unproven. We examine inaccuracies in the estimation of SARs and β‐diversity parameters that emerge from imperfect detection, and whether such errors can be mitigated using a non‐parametric diversity estimator (iNEXT.3D) and Multi‐Species Occupancy Models (MSOMs). We simulated 28,350 sampling regimes of 2835 fragmented communities, varying the mean and standard deviation of species detection probabilities, and the number of sampling repetitions. We then quantified the bias, accuracy, and precision of derived estimates of model coefficients for SARs and the effects of patch area on β‐diversity (pairwise Sørensen similarity). Imperfect detection biased estimates of all evaluated parameters, particularly when mean detection probabilities were low, and there were few sampling repetitions. Observed counts consistently underestimated species richness and SAR *z*‐values, and overestimated SAR c‐values; iNEXT.3D and MSOMs only partially resolved these biases. iNEXT.3D provided the best estimates of SAR z‐values, although MSOM estimates were generally comparable. All three methods accurately estimated pairwise Sørensen similarity in most circumstances, but only MSOMs provided unbiased estimates of the coefficients of models examining covariate effects on β‐diversity. Even when using iNEXT.3D or MSOMs, imperfect detection consistently caused biases in SAR coefficient estimates, calling into question the robustness of previous SAR studies. Furthermore, the inability of observed counts and iNEXT.3D to estimate β‐diversity model coefficients resulted from a systematic, area‐related bias in Sørensen similarity estimates. Importantly, MSOMs corrected for these biases in β‐diversity assessments, even in suboptimal scenarios. Nonetheless, as estimator performance consistently improved with increasing sampling repetitions, the importance of appropriate sampling effort cannot be understated.

## Graphical abstract

Using simulations, we show that imperfect detection substantially biases estimates of the coefficients of species‐area relationships and pairwise β‐diversity models. Although the use of both iNEXT.3D and Multi‐Species Occupancy Models improved observed biases in SAR coefficient estimates, neither method fully resolved them. Importantly, however, MSOMs consistently corrected for imperfect detection in β‐diversity estimates, providing highly accurate estimates of pairwise β‐diversity trends even in sub‐optimal conditions.

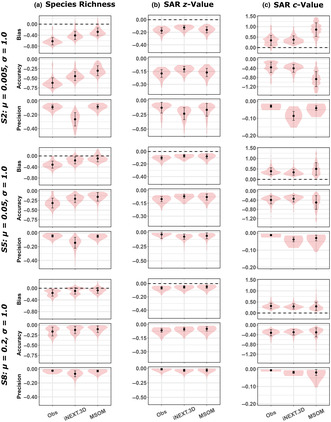

## 1 INTRODUCTION

A major aim in ecology centres on identifying relationships between the spatial configuration of habitat patches and their biotic assemblages (Ewers et al., 2010; MacArthur & Wilson, 1963). Researchers have typically addressed this issue via the use of species‐area relationships (henceforth ‘SARs’), used to assess how local species richness declines with habitat patch area (MacArthur & Wilson, 1963), and analyses of species composition (beta‐diversity), which provide additional insight into how species are distributed throughout disturbed landscapes (Banks‐Leite et al., 2012; Socolar et al., 2016). Historically, estimates of both species richness and β‐diversity indices have been derived from observed species counts (Dorazio et al., 2010; MacKenzie & Royle, 2005). However, observed counts usually represent only a subset of the true community (Colwell et al., 2004), and failure to account for this can lead to significant biases in derived community metrics and subsequent community models (Gwinn et al., 2015; McNew & Handel, 2015). Correcting for imperfect detection is thus widely recognized as vital for making robust conservation and management decisions within human‐modified landscapes (Banks‐Leite et al., 2014; Zipkin et al., 2009). However, the importance of correcting for imperfect detection has received surprisingly little attention in studies aiming to quantify SARs and beta‐diversity trends.

Estimating species richness has been a longstanding issue (Fisher et al., 1943; Gwinn et al., 2015), and many statistical methods exist to estimate true richness from observed species counts. For instance, the Chao family of non‐parametric estimators, including the iNEXT.3D programme (Chao et al., 2021), are widely used to estimate asymptotic species richness based on rarefaction and/or the frequency of rare species within a sample (e.g., Mendenhall et al., 2014; Palmeirim et al., 2021), and have also been expanded to estimate detection‐corrected β‐diversity indices (iNEXT.beta3D; Chao et al., 2023). While addressing imperfect detection, the Chao/iNEXT.3D estimators do not consider the influence of covariates on species occurrence or detection (MacKenzie et al., 2005). Hierarchical Multi‐Species Occupancy Models (MSOMs; e.g., Jones et al., 2021; Semper‐Pascual et al., 2021) offer an alternative approach that can explicitly model occurrence and detection probabilities of both observed and unobserved species (Kéry & Royle, 2008; Zipkin et al., 2009). Researchers may then derive asymptotic estimates of species richness and β‐diversity from the species‐specific occurrence estimates (Broms et al., 2015), providing a directly comparable alternative to the Chao/iNEXT.3D estimators.

When analysing spatial patterns in biodiversity such as SARs and β‐diversity relationships, researchers should seek to correct for imperfect detection using methods that accurately uncover true variation in assemblage structure (Iknayan et al., 2014). However, few studies have examined how effective currently available techniques are for this purpose. Accounting for imperfect detection can dramatically alter insights. For example, Palmeirim et al. (2021) found that SAR slopes increased twofold when observed counts were replaced with iNEXT (the predecessor to iNEXT.3D; Hsieh et al., 2016) richness estimates in a community of Amazonian snakes, a group with infamously low detection probabilities (Durso et al., 2011; Fraga et al., 2014). However, statistical estimators, including Chao/iNEXT. 3D and MSOMs, tend to perform poorly when sample sizes are small and species detection probabilities are low (McNew & Handel, 2015; Tingley et al., 2020), and it is thus possible that the dramatic changes in SAR slopes observed by Palmeirim et al. (2021) represents an artefact of estimation error (Type I Error), rather than underlying patterns of species richness (Gwinn et al., 2015). Further investigation is thus required to determine how sampling effort influences the estimation of biodiversity trends within fragmented landscapes, and the circumstances where statistical estimators may be able to reliably correct for deficiencies in sampling (Montgomery et al., 2021).

Previous studies have assessed the accuracy of MSOM and Chao/iNEXT.3D richness estimates using simulations and subsets of exhaustive empirical data, where the true values of community properties are known (McNew & Handel, 2015, Tingley et al., 2020). However, no previous study has examined the relative performance of MSOMs and Chao/iNEXT.3D for SAR estimation. This evaluation is urgently needed, as the richness estimates that underpin SARs are known to be highly sensitive to species' detection probabilities (McNew & Handel, 2015, Tingley et al., 2020), the underlying species abundance distribution (Gwinn et al., 2015; Rajakaruna et al., 2016), and sampling design (MacKenzie & Royle, 2005).

Imperfect detection has also been recognized as an issue in β‐diversity analyses (Dorazio et al., 2010; Nilsson & Nilsson 1982, 1983) and frequently used β‐diversity indices have previously been shown to be sensitive to undersampling (Cardoso et al., 2009; Roden et al., 2018). Estimating β‐diversity in the absence of complete species catalogues may be even more challenging than estimating species richness: while richness estimators need only estimate the number of species occurring within a single assemblage, the estimation of β‐diversity requires estimating both the number and identity of species in two or more assemblages (Barwell et al., 2015; Cardoso et al., 2009). Indeed, via a case study on a long‐term butterfly monitoring dataset (Swiss Biodiversity Monitoring Program), Dorazio et al. (2010) demonstrated that observed species counts tend to substantially underestimate pairwise Jaccard similarity compared to MSOMs. However, to our knowledge, there has been no assessment of the comparative performance of MSOMs and non‐parametric β‐diversity estimators (the latter of which are more accessible to most ecologists), and there is a need to determine whether the findings of Dorazio et al. (2010) are generalizable to other settings, e.g., fragmentation ecology.

Here, we use simulations to explore the impacts of imperfect detection on typical field study designs for SAR and β‐diversity estimation. We assess the comparative performance of observed species counts, an abundance‐based Chao estimator (iNEXT.3D/iNEXT.beta3D; Chao et al., 2021, 2023) and MSOMs to estimate patch‐level species richness, the slope and intercept of species‐area relationships (SARs), pairwise β‐diversity between each pair of patches, and the slope and intercept of the relationship between pairwise differences in patch area and pairwise β‐diversity. We evaluate the sensitivity of estimators to variation in species detectabilities, and the sampling design used to assess communities. We then make recommendations on the relative effectiveness of each estimator and consider the circumstances in which it is preferable to use either iNEXT.3D, MSOMs, or observed species counts to assess patterns of species richness and/or β‐diversity.

## 2 METHODS

### 2.1 Community parameters of interest

We defined a novel framework to simulate realistic communities and sampling designs that are typically applied in SAR and β‐diversity research, focusing on biodiversity patterns across fragmented landscapes. While MSOMs can only incorporate data on species incidence (i.e., detection/non‐detection; Kéry & Royle, 2008), there are both incidence and abundance‐based versions of iNEXT.3D (Chao et al., 2021, 2023). We therefore opted to simulate abundance‐based communities and sampling procedures, and then collapse the species counts into incidence data for occupancy modelling.

Using our simulated landscape communities, we assessed the extent to which imperfect detection causes inaccuracies/biases in estimates of a total of six community parameters (i.e., differences between the true parameter values and estimates derived from observed species counts), and whether MSOMs and iNEXT.3D can correct for such errors. Here, we outline the formulae used to calculate the true values of each community parameter:

- *Parameter 1*: Patch‐level species richness, defined as the number of species that occur within each habitat patch p:
$$S_{p}=\sum \left[N_{p,i}>0\right]$$

where $N_{p,i}$ is a vector containing the true abundance of each species i in patch p.

- *Parameters 2 and 3*: The intercept (*c*‐value) and slope (*z*‐value) of a log–log (power) model of the relationship between patch‐level species richness $S_{p}$ and patch area ${\text{Area}}_{p}$ (i.e., SAR; Rosenzweig, 1995):
$$\log_{n}\left(S_{p}\right)=c+z\times \log_{n}\left({\text{Area}}_{p}\right)$$
- *Parameter 4*: Pairwise Sørensen similarity between the communities of each pair of patches:
$${\mathrm{Sør}}_{a,b}=\frac{2\;\left|A\cap B\right|}{\left|A\right|+\left|B\right|}$$

where A and B are the true species occurrence records for patches a and b, respectively.

- *Parameters 5 and 6*: The intercept ($\gamma_{0}$) and slope ($\gamma_{1}$) of the relationship between pairwise Sørensen similarity and the log_n_(*x* + 1) transformed pairwise difference in patch area ${\text{ΔArea}}_{a,b}$:
$$\text{Logit}\left(Sør_{a,b}\right)=\gamma_{0}+\gamma_{1}\times \log_{n}\left({\text{ΔArea}}_{a,b}+1\right)$$
  As the Sørensen similarity index is constrained to values between 0 and 1, we modelled pairwise β‐diversity using a logit link.

As all incidence‐based indices of overall compositional similarity (e.g., Jaccard, Sørensen) are calculated as a function of the species richness of individual sites and the number of species shared between sites (Baselga, 2010), estimation accuracy should not vary between indices derived from the same dataset. We therefore assessed the impact of imperfect detection on β‐diversity estimation using only the pairwise Sørensen similarity index (Sørensen, 1948).

#### 2.1.1 Simulating fragmented landscape communities

Each simulated landscape featured 25 habitat patches of varying area, with the smallest and largest patches pre‐assigned areas of 25 and 20,000 Ha, respectively. The areas of the remaining 23 patches were drawn from a four‐parameter beta distribution:

$${\text{Area}}_{p}\sim \text{Beta}4\left(\alpha =1,\beta =4,\min=25,\max=20,000\right)$$

Here, ${\text{Area}}_{p}$ is the area of patch p, α and β are the shape parameters of the four‐parameter beta distribution, and min and max are the smallest and largest possible patch areas, respectively. These parameters generated a negative power law relationship between frequency and area, as is typical of real‐world fragmented tropical forest landscapes (Taubert et al., 2018). The generated patch areas also covered ~3.15 orders of magnitude, encompassing the range typically sampled in habitat fragmentation studies (mean ± SD = 2.75 ± 0.08; Watling & Donnely, 2006).

We then generated the landscape metacommunity according to a log‐normal species abundance distribution (mean = 650, SD = 3), implemented using the ‘sim_sad’ function of the ‘*mobsim’* R package (May et al., 2018; R Core Team, 2023). This enabled us to control: (1) the number of species and (2) the number of individuals. We set the number of simulated species within each landscape to 200. To ensure a minimum viable landscape‐level population size of each species, we first set the number of individuals to equal the total area (Ha) of all patches in the landscape, and subsequently multiplied the resultant species abundances by 500 to give the final metacommunity. Therefore, the minimum number of individuals of each species within a landscape was 500, and overall population density was 500 individuals per Ha. We assumed population density to be consistent throughout each landscape, and the carrying capacity K of each patch p was thus:

$$K_{p}={\text{Area}}_{p}\times 500$$

Next, we generated area responses for each species ($\beta_{\text{Area},i}$) using draws from species‐specific four‐parameter beta distributions:

$$\begin{array}{c} \beta_{\text{Area},i}\sim \text{Beta}4\left(\alpha =\alpha_{i},\beta =5,\min=0,\max=5\right) \end{array}$$

Here, $\alpha_{i}$ is a species‐specific value of the first shape parameter α, β is the second shape parameter (consistent across species), and min and max are the lowest and highest possible values of $\beta_{\text{Area},i}$, respectively. For each simulated community, we sequentially generated $\alpha_{i}$ for each species across the range 0.1 to $\alpha_{\max}$, in inverse proportion to the species' simulated abundances. This meant that species with lower landscape‐level abundance tended to have area responses of greater magnitude, as is apparent in real‐world fragmented communities (Franzén et al., 2012; Keinath et al., 2017). To simulate natural variation in the area‐related structuring of fragmented communities, we varied $\alpha_{\max}$ between simulated communities ($\alpha_{\max}$ = 4, 8 or 12), with greater $\alpha_{\max}$ values yielding communities with greater SAR *z‐*values (i.e., steeper slope). Furthermore, as patch area responses are rarely uniformly positive (Jones et al., 2021; Noble et al., 2023), we randomly selected 1/8th of the species in each community and multiplied their area response by −1, thereby yielding a negative area response.

**FIGURE 1:**
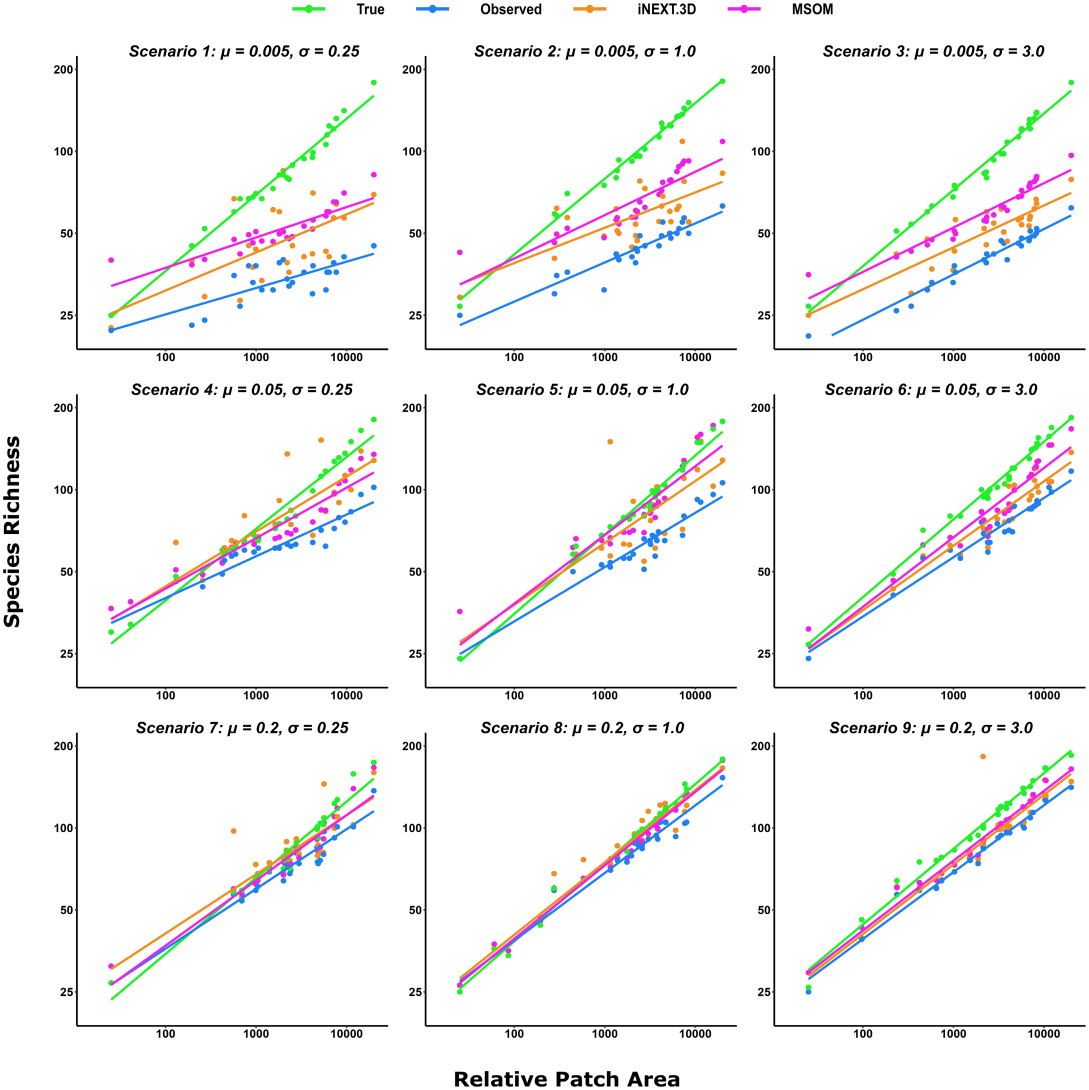
Examples of species‐area relationships (SARs) constructed using the true richness values (True) and richness estimates from observed species counts (Observed), the abundance‐based iNEXT.3D estimator (iNEXT.3D), and Multi‐Species Occupancy models (MSOM). The results from a single sampling process within a single simulated landscape are shown for each of the nine possible detection scenarios. In each depicted instance, six repeat sampling visits were simulated within each patch (*V* = 6) and the maximum α parameter of the species‐specific area response distributions was set to eight ($\alpha_{\max}$= 8). Scenarios are presented in order of increasing mean (*μ*; top to bottom) and standard deviation (*σ*; left to right) of the detection probability hyperparameter.

Using the generated species area responses, we then determined the probability of an individual of each species i being assigned to each patch p, using the functions:

$$\begin{array}{c} W_{p,i}=\exp\left(\beta_{\text{Area},i}\times \log_{n}\left({\text{Area}}_{p}\right)\right) \end{array}$$

$$\begin{array}{c} \phi_{p,i}=\frac{W_{p,i}}{\sum_{p=1}^{25}W_{p,i}} \end{array}$$

where $W_{p,i}$ is the assignment weight and $\phi_{p,i}$ is the assignment probability (i.e., $W_{p,i}$ scaled to sum to one). We iteratively assigned species individuals to patches according to the species' assignment probabilities (i.e., weighted random sampling). To ensure a minimum ‘viable’ patch‐level population of each species, individuals were assigned to groups of 100. When the population carrying capacity $K_{p}$ of a patch was reached, the patch was removed from the potential assignment pool. This yielded communities with realistic power model (log–log) SARs (Rosenzweig, 1995; see Figure 1), with the *z‐*values ranging between 0.137 and 0.345, roughly encompassing the range typically observed in real‐world landscapes (Matthews et al., 2016).

#### 2.1.2 Simulating sampling procedures

To simulate imperfect sampling, we drew individual‐level detection probabilities for each species $\theta_{i}$ from community‐level hyperparameter distributions:

$$\begin{array}{c} \text{Logit}\left(\theta_{i}\right)\sim N\left(\text{Logit}\left(\mu \right),\sigma \right) \end{array}$$

where μ is the mean and σ the standard deviation of the detection probability hyperparameter for a given landscape community. To investigate the influence of variation in detection probabilities on SAR and β‐diversity estimation, we varied μ and σ among simulated communities (see Table 1).

**TABLE 1:** The nine detection scenarios were simulated, varying the mean (μ) and standard deviation (σ) of the detection probability hyperparameter of the sampled communities, from which individual‐level species detection probabilities were drawn.

Scenario ID	Mean detection probability (*μ*)	Standard deviation of detection probability (*σ*)
S1	0.005	0.25
S2	0.005	1.0
S3	0.005	3.0
S4	0.05	0.25
S5	0.05	1.0
S6	0.05	3.0
S7	0.2	0.25
S8	0.2	1.0
S9	0.2	3.0

*Note*: We simulated 35 landscape communities and 350 sampled datasets (i.e., 10 sampling processes were simulated for each landscape community) using every possible combination of detection probability μ and σ, three levels of repeat sampling visits to each site (V: 3, 6, or 12), and three values for the species‐specific area response distributions ($\alpha_{\max}$: 4, 8, or 12). There were thus 81 possible parameter combinations, resulting in a total of 2,835 simulated landscape communities and 28,350 simulated sampling datasets.

Fragmentation ecologists usually sample only a spatial subset of each habitat patch, and scale sampling effort with patch area (Azovsky, 2011, Rybicki & Hanski, 2013). To emulate this, we simulated sampling from a set of 500 m transects within each patch, each with a 50‐m search radius (i.e., one transect = 5.93 ha). We varied the number of transects placed according to patch area so that the total area of each patch sampled $\pi_{p}$ increased roughly proportionally to patch size (Appendix S1: Table S1). Then, we calculated the number of individuals of each species in each patch that were available for sampling $n_{p,i}$ as the proportion of all individuals in the patch $N_{p,i}$ present within $\pi_{p}$, assuming constant within‐patch densities and rounding to the nearest integer:

$$n_{p,i}=\text{Round}\left(N_{p,i}\times \pi_{p}\right)$$

Next, we simulated replicate surveys of each patch as a series of binomial trials, where the number of individuals X of species s observed in patch p on visit v was determined by their species‐specific detection probabilities. Thus:

$$\begin{array}{c} X_{p,i,v}\sim \text{Binomial}\left(n_{p,i},\theta_{i}\right) \end{array}$$

As previous studies have shown that the accuracy of both MSOMs and Chao estimators is correlated with sample size (McNew & Handel, 2015; Tingley et al., 2020), we varied the total number of replicate surveys V at each site among simulated communities (Table 1).

#### 2.1.3 Simulation repetitions

We simulated 10 communities using each possible combination of *μ* (0.005, 0.05, 0.2), *σ* (0.25, 1.0, 3.0), $\alpha_{\max}$ (4, 8, 12), and V (3, 6, 12) (total possible combinations = 81). To determine the extent to which stochastic variation in sampling may affect estimator performance (Walther & Moore, 2005), we generated 10 separate sampled datasets for each simulated landscape community, using the same parameters but different seed values. Therefore, we simulated a total of 2835 communities and 28,350 sampled datasets (Table 1).

### 2.2 Estimating richness and β‐diversity

#### 2.2.1 Observed species counts

To quantify the effects of imperfect detection on species richness and estimates, we first calculated patch‐level species richness and pairwise Sørensen similarity based on the observed species counts pooled across all sampling repetitions (i.e., using $X_{p,i}$ in place of the true abundance values in the formulae outlined in Section 2.1).

#### 2.2.2 iNEXT.3D/ iNEXT.3betaD

We generated iNEXT.3D estimates of patch‐level species richness and Sørensen similarity using the ‘*iNEXT.3D*’ and ‘*iNEXT.beta3D*’ R packages, respectively (Chao et al., 2021, 2023, R Core Team, 2023). We used the abundance‐based versions of iNEXT.3D and iNEXT.beta3D, and applied these to the observed species counts pooled across all sampling repetitions. We took the mean estimated values as point estimates of richness and Sørensen similarity and used these for all further analyses.

#### 2.2.3 MSOMs

To fit MSOMs, we generated an incidence matrix $Y_{p,i}$, indicating the number of sampling occasions where each species i was detected in each patch p. We augmented the incidence matrix with double the number of unobserved species (i.e., all 0 observation records) as observed species (Kéry & Royle, 2008), for a total number of possible species M. The model likelihood took the form:

$$\begin{array}{c} \omega_{m}\sim \text{Bernoulli}\left(\Omega \right) \end{array}$$

$$\begin{array}{c} z_{p,m}\sim \text{Bernoulli}\left(\omega_{m}\times \psi_{p,m}\right) \end{array}$$

$$\begin{array}{c} y_{p,m}\sim \text{Binomial}\left(V,z_{p,m}\times \vartheta_{p,m}\right) \end{array}$$

$$\begin{array}{c} \text{Logit}\left(\psi_{p,m}\right)=\delta_{0,m}+\delta_{1,m}\times {\text{Area}}_{p} \end{array}$$

$$\begin{array}{c} \text{Logit}\left(\vartheta_{p,m}\right)=\lambda_{0,m} \end{array}$$

Here, $\omega_{m}$ is a latent variable indicating whether each species m in the augmented dataset was truly present in the overall community; Ω is the probability of a species being present in the overall community; $z_{p,m}$ is a binary variable indicating whether species m truly occurred in patch p; $\psi_{p,m}$ and $\vartheta_{p,m}$ are the estimated occurrence and detection probabilities of species m in patch p, respectively; $\delta_{0,m}$ and $\lambda_{0,m}$ are the species‐specific occurrence and detection model intercepts, respectively; and $\delta_{1,m}$ is the species‐specific effect of patch area on occurrence probability.

We specified a joint‐bivariate normal prior for the occurrence $\delta_{0,m}$ and detection $\lambda_{0,m}$ model intercepts (Zipkin et al., 2009) and drew all species‐level parameters from community‐level hyperparameter distributions (Dorazio et al., 2006). We used beta priors (1,1) for intercept hyperparameter means, normal priors (0,1) for slope hyperparameter means, uniform priors (0,5) for hyperparameter variance components, and an approximation of Link's scale prior for the community inclusion parameter Ω (Link, 2013).

We fitted MSOMs using the ‘nimble’ R package (de Valpine et al., 2017; R Core Team, 2023). Inference was made from 4 chains of 50,000 Markov Chain Monte Carlo (MCMC) iterations, each with a burn‐in of 10,000 and a thinning factor of 20, resulting in a total of 8000 MCMC iterations being retained from each model. These MCMC parameters were sufficient to achieve acceptable chain convergence in preliminary testing, assessed according to the Gelman‐Rubin Diagnostic, where values of <1.05 were taken to indicate proper convergence (Gelman & Rubin, 1992). Full model specification is available in Appendix S2.

Using the species occurrence estimates $z_{p,m}$ from each model iteration, we then estimated species richness for each patch p:

$${\hat{S}}_{p}=\sum z_{p,1:M}$$

And Sørensen similarity between each pair of patches (a and b):

$${\hat{Sør}}_{a,b}=\frac{2\left|z_{a,1:M}\cap z_{b,1:M}\right|}{\left|z_{a,1:M}\right|+\left|z_{b,1:M}\right|}$$

We then calculated the posterior mean values of patch‐level species richness and pairwise Sørensen similarity across all iterations and used these as point estimates for all further analyses.

### 2.3 Estimating SARs and effects of area on β‐diversity

For each simulated dataset, we constructed log–log (power model) SARs (Rosenzweig, 1995) using the richness estimates derived from observed species counts, and iNEXT.3D and MSOM (i.e., substituted the richness estimates into the SAR formula in Section 2.1). To model the effect of patch area on pairwise β‐diversity, we constructed a pairwise environmental distance matrix containing the absolute difference in area between each pair of patches. We then modelled the effect of the log‐transformed distance matrix values (log_*n*_(*x* + 1)) on the Sørensen similarities derived from observed species counts, and iNEXT.3D and MSOMs using a generalized linear model with a logit link (i.e., substituted the Sørensen similarity estimates into the formula in Section 2.1). In all instances, the coefficients of SARs (*c*‐value and *z*‐value) and β‐diversity models (intercept and slope) were estimated using Maximum Likelihood Estimation, and we used the mean coefficient estimates (point estimates) for all further analysis.

### 2.4 Assessing estimator performance

We assessed the performance of observed species counts, MSOMs and iNEXT.3D, as estimators of each of the six community parameters using three criteria (Walther & Moore, 2005). In all cases, the criteria were calculated using the parameter estimates derived based on the difference between the true parameter values (see Section 2.1) and the corresponding parameter estimates from each of the 10 replicate datasets from each simulated landscape:

1. Bias – The mean of the signed differences between the point estimates and true values. For site‐level richness, we divided bias values by the true richness, to quantify percentage differences (i.e., scaled mean error).
2. Accuracy – The mean of the absolute differences between the point estimates and true values, multiplied by −1. As with bias, we divided accuracy values of site‐level richness estimates by the true richness.
3. Precision – The standard deviation of the point estimates divided by the absolute mean of the point estimates (i.e., coefficient of variation), multiplied by −1.

## 3 RESULTS

### 3.1 Species richness and SARs

**FIGURE 2:**
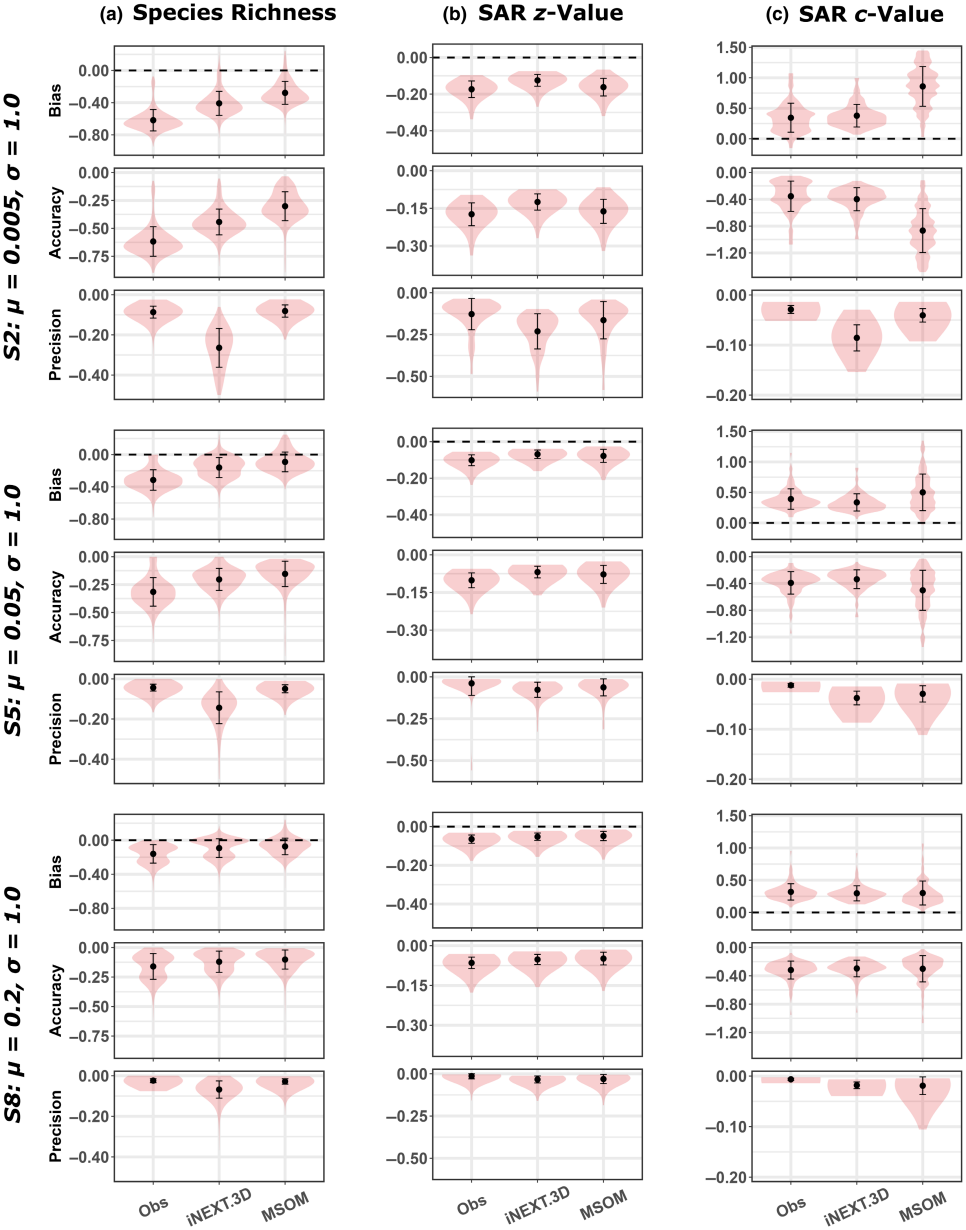
Estimator performance for (a) site‐level species richness, (b) the slope (*z*‐value) of species‐area relationships (SAR), and (c) the intercept (*c*‐value) of SARs, from representative scenarios where the standard deviation of the detection probability was set to the intermediate value (i.e., *σ* = 1.0) and six repeat sampling visits were simulated for each site. Estimates were derived from observed species counts (Obs), the abundance‐based iNEXT.3D estimator (iNEXT.3D) and Multi‐Species Occupancy models (MSOM), presented in order of increasing mean detection probability (*μ*). Bias and accuracy of site‐level richness estimates are presented in units of percentage difference from the true richness. Results from other simulated scenarios are shown in Appendix S1.

MSOMs and iNEXT.3D always provided more accurate and less biased estimates of species richness than observed species counts. MSOMs consistently provided the least biased and most accurate richness estimates, though all three methods tended to underestimate species richness to some degree (Figure 2a; Appendix S1: Figure S1). In all simulated scenarios, iNEXT.3D richness estimates were considerably less precise than estimates from MSOMs and observed counts, among which precision was comparable. The accuracy, precision, and bias of richness estimates improved with increased sampling effort (i.e., number of repeat samples) in all cases, though MSOM estimates showed the lowest sensitivity to sampling effort (Appendix S1: Figure S1). Similarly, MSOMs were the least sensitive of the three methods to variation in the mean and standard deviation of species detection probabilities (Figure 2a; Appendix S1: Figure S1).

All three methods consistently underestimated SAR *z*‐values in the presence of imperfect detection (Figures 1 and 2b; Appendix S1: Figure S2). iNEXT.3D provided slightly less biased and more accurate *z*‐value estimates than the other methods when mean detection probabilities were lowest (*μ* = 0.005). At higher mean detection probabilities (*μ* = 0.05 or 0.2), the bias and accuracy of MSOM and iNEXT.3D z‐value estimates were largely comparable, on average. Both MSOMs and iNEXT.3D tended to provide more accurate and less biased estimates of SAR *z*‐values than observed species counts, apart from when the standard deviation of species detection probabilities was highest (*σ* = 3.0), in which case accuracy and bias were comparable among the three methods (Appendix S1: Figure S2). All three estimators also tended to overestimate SAR *c*‐values (Figures 1 and 2c, Appendix S1: Figure S3). However, observed counts provided largely unbiased *c*‐value estimates when mean detection probability was lowest (*μ* = 0.005) and the standard deviation highest (*σ* = 3.0; Appendix S1: Figure S3).

Estimates of both SAR coefficients tended to become more accurate and less biased as mean detection probabilities increased, regardless of the estimator used. This effect was most apparent in MSOM *c*‐value estimates, which were considerably more biased than estimates from observed counts and iNEXT.3D when the mean detection probability was low (*μ* = 0.005), but often outperformed the other estimators when mean detection probability across the community was high (*μ* = 0.2), except for cases in which the standard deviation of detection probabilities was also high (*σ* = 3.0; Appendix S1: Figure S3). Greater variability in detection probabilities consistently resulted in reduced bias and increased accuracy for estimates of both SAR coefficients derived from observed counts, iNEXT.3D and, to a lesser extent, MSOMs (Appendix S1: Figures S2, S3). However, at higher mean detection probabilities, increases in the standard deviation of detection probabilities led MSOM estimates of both SAR coefficients to become more biased and less accurate (Appendix S1: Figures S2, S3).

Precision was generally highest for SAR coefficient estimates derived from observed counts. iNEXT.3D consistently provided the least precise estimates of SAR *z*‐values (Figure 2b,c; Appendix S1: Figures S2, S3) and the least precise estimates of SAR *c*‐values at all but the highest mean detection probability (*μ* = 0.2), where MSOM *c*‐value estimates tended to be slightly less precise. The precision of estimates of both SAR coefficients increased with increasing mean detection probability regardless of the method used, although the magnitude of this variation was greatest for iNEXT.3D.

#### 3.1.1 Pairwise β‐diversity

**FIGURE 3:**
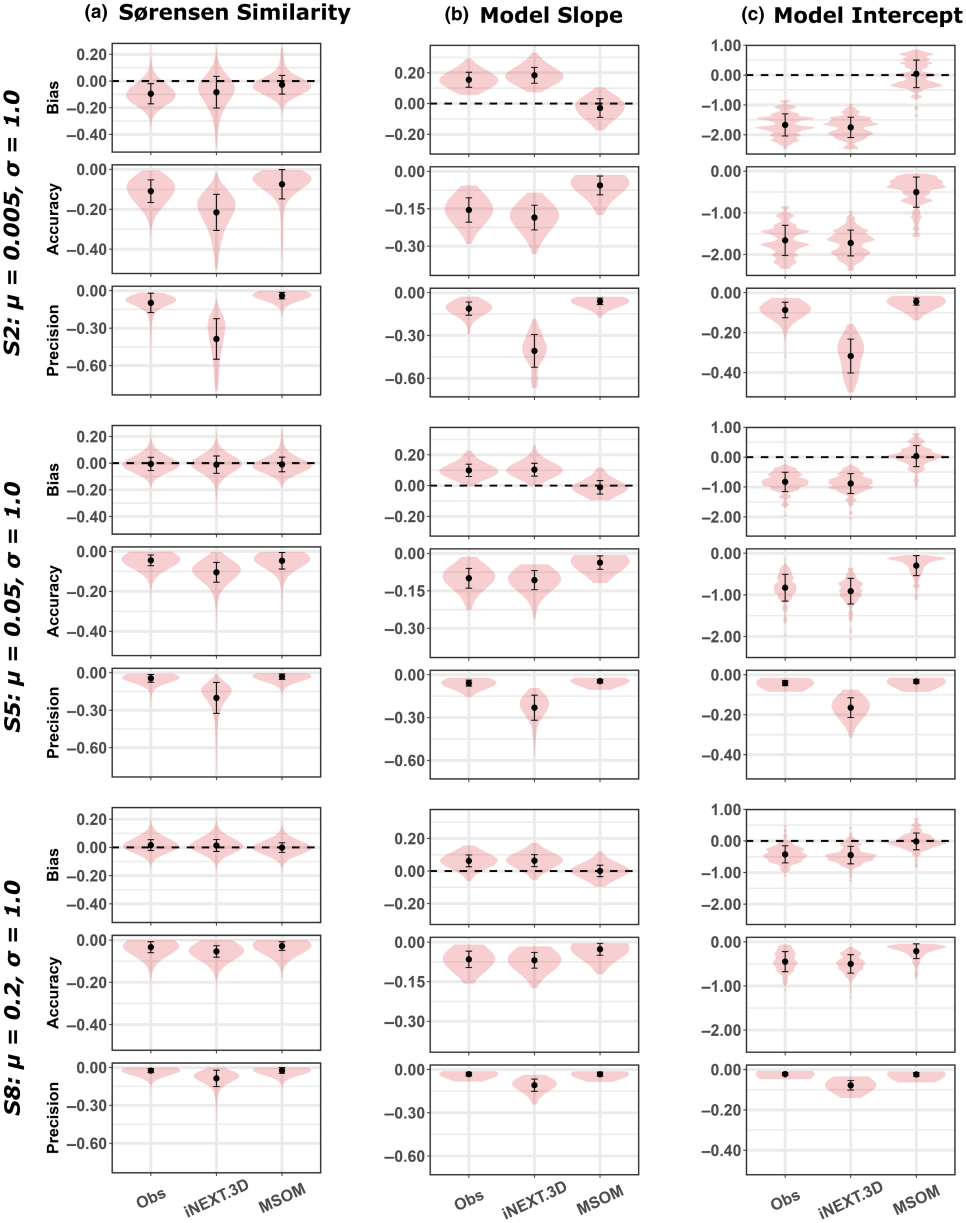
Estimator performance for β‐diversity metrics, measured as (a) pairwise Sørensen similarity, (b) the slope of models relating pairwise Sørensen similarity to patch area, and (c) the intercept of pairwise Sørensen similarity models, shown for representative scenarios with intermediate standards deviations of the detection probability (*σ* = 1.0) and six repeat sampling visits for each site. Estimates were derived from observed species counts (Obs), the abundance‐based iNEXT.beta3D estimator (iNEXT.3D) and Multi‐Species Occupancy models (MSOM). Bias and accuracy are presented in their native units. Simulation scenarios are presented in order of increasing mean detection probability (*μ*), from top to bottom. For results from all simulated scenarios, see Appendix S1.

All three methods estimated pairwise Sørensen similarity with reasonable accuracy and bias in most scenarios, though MSOMs provided the most accurate, least biased, and most precise estimates in most scenarios. Interestingly, iNEXT.3D estimates tended to be more biased, less accurate, and less precise than Sørensen estimates derived from observed counts in almost all scenarios (Figure 3a; Appendix S1: Figure S4).

In scenarios where community mean detection probability was lowest (*μ* = 0.005), all methods underestimated Sørensen similarity, though estimates became less biased and more accurate with increases in sampling repetitions and detection probability standard deviations (Appendix S1: Figure S4). However, at higher mean detection probabilities, variation in sampling repetitions and the standard deviation of detection probabilities had negligible impacts on Sørensen estimate accuracy. The precision of Sørensen estimates increased with the mean of detection probabilities and the number of sampling repetitions, regardless of the method used. However, the magnitude of sensitivity to sampling and detection variation was considerably greater for iNEXT.3D estimates than for observed species counts and MSOMs, the latter being relatively robust to variation in sampling and detection variation in terms of bias, accuracy, and precision (Appendix S1: Figure S4).

#### 3.1.2 β‐Diversity model coefficients

Under all scenarios, MSOMs provided substantially more accurate and less biased estimates of the slope and intercept of models for area effects on pairwise Sørensen similarity, relative to observed counts and iNEXT.3D (Figure 3b,c; Appendix S1: Figures S5, S6). Indeed, MSOMs consistently provided unbiased estimates in all simulated scenarios (mean bias values ≈0). MSOM accuracy varied minimally with increases in the number of sampling repetitions, and while MSOM coefficient estimates tended to be more accurate when the mean and standard deviation of detection probability were higher, the magnitude of this variation was only slight (Appendix S1: Figures S5, S6).

In contrast, estimates of Sørensen model coefficients derived from observed counts and iNEXT.3D tended to become substantially less accurate and more biased with fewer sampling repetitions and lower mean detection probabilities and were never comparable to MSOM estimates. Variation in the standard deviation of species detection probabilities had minimal impact on the bias and accuracy of coefficient estimates derived from observed counts and iNEXT.3D. In all scenarios, iNEXT.3D provided the least precise estimates of both β‐diversity model coefficients. Precision tended to increase with mean detection probability and the number of sampling repetitions, regardless of the method used. However, this variation was most marked for iNEXT.3D coefficient estimates and least apparent for MSOM estimates (Appendix S1: Figures S5, S6).

## 4 DISCUSSION

We show that imperfect detection substantially biases estimates of SAR parameters, pairwise β‐diversity (Sørensen similarity), and β‐diversity model coefficients. Importantly, we found that SAR *z‐*values were consistently underestimated, and SAR *c*‐values overestimated, even when statistical approaches were used to account for imperfect detection (i.e., iNEXT.3D or MSOMs). This highlights an important limitation in SAR research, potentially calling into question the accuracy of assessments of the impacts of habitat fragmentation on species richness. In empirical settings, a systematic negative bias in SAR z‐value estimates would result in underestimates of the number of local species extinctions that result from increasing habitat fragmentation, potentially leading to recommendations of minimum habitat patch sizes below those required for the persistence of species with large area requirements (Cam et al., 2002; Montgomery et al., 2021). Furthermore, although observed species counts were generally able to provide accurate pairwise Sørensen similarity estimates, this did not translate into accurate estimates of β‐diversity model coefficients, and such inaccuracies were only worsened by the application of iNEXT.3D. Encouragingly, MSOMs consistently provided accurate and unbiased estimates of pairwise Sørensen similarity, and both β‐diversity model coefficients, even when detection probabilities, were low. The superior performance of MSOMs relative to the other methods highlights how failure to account for covariate effects (e.g., patch area) on species occupancy can substantially bias assessments of pairwise β‐diversity trends (Dorazio et al., 2010; Iknayan et al., 2014).

### 4.1 Richness and SARs

Rarely are all species in a community detected by sampling (Longino et al., 2002), and species richness estimates derived from observed counts are thus expected to be negatively biased (Kéry & Royle, 2008; McNew & Handel, 2015). However, our results suggest that iNEXT.3D and MSOMs still routinely underestimate species richness under sampling designs typically applied in fragmentation ecology research and consequently struggle to generate accurate estimates of SAR parameters. In a previous study focussing on richness estimation, McNew and Handel (2015) found that the asymptotic Chao estimator (Chao, 1984) consistently overestimated richness, while MSOM richness estimates were generally unbiased. Discrepancies between these findings and our own likely result from the simulated sampling designs: McNew and Handel (2015) simulated spatially complete sampling of their target areas, whereas we only simulated sampling of a spatial subset of each habitat patch.

Spatially exhaustive sampling is rarely feasible in empirical ecological research, particularly fragmentation ecology (Eigenbrod et al., 2011; Pasher et al., 2013), and previous research has highlighted that the portion of a habitat patch that is sampled may often be of insufficient size to encounter the first individual of species with low abundance and/or spatially aggregated distributions (Cam et al., 2002; He & Hubbell, 2011). We deliberately sought to emulate this in our simulation framework, calculating the number of species individuals that were available for sampling within each patch as a function of the proportional area sampled (see Section 2.1.2). Therefore, species with low patch‐level abundance would often be unavailable for sampling, as their abundance within the target area would be rounded to zero. In this way, our simulation framework adds an additional source of imperfect detection compared to previous, similar studies that have simulated complete spatial sampling (e.g., Guillera‐Arroita et al., 2019; McNew & Handel, 2015; Tingley et al., 2020). Importantly, our findings suggest that neither observed species counts, MSOMs or iNEXT.3D, can fully account for this incomplete spatial sampling, as they routinely underestimated species richness. Furthermore, inspecting the relationship between patch area and bias in richness estimates showed that while iNEXT.3D and observed counts typically provided relatively unbiased estimates for small habitat patches, where the proportion of each patch sampled was highest, richness estimates derived from all three methods exhibited considerable negative biases as patch size increased, and the proportion of each patch sampled decreased (Appendix S1: Table S1, Figure S7). This is perhaps unsurprising, given that the proportion of individuals captured will tend to increase with the proportional area of a patch that is sampled, and that previous research has shown that Chao and MSOM richness estimates improve with increases in other aspects of sampling effort (Chao et al., 2009; McNew & Handel, 2015; Tingley et al., 2020). Further research seeking to determine the minimum proportion of a target area that must be sampled to accurately estimate species richness, both when using observed species counts and statistical richness estimators, should thus constitute a vital next step in optimizing ecological study design (Cam et al., 2002; He & Hubbell, 2011).

Interestingly, MSOMs tended to overestimate richness of the smallest habitat patches and underestimate richness of the largest habitat patches, with the magnitude of these biases being comparable (Appendix S1: Figure S7), in turn explaining why MSOMs still performed relatively poorly as estimators of SAR *c*‐values. This may, in part, be explained by the fact that MSOMs draw species‐level covariate responses (slopes) from community‐level hyperparameters, and for unobserved species these slopes thus tend toward the hyperparameter mean, in a process termed shrinkage (Everitt, 2002). Given that we simulated species with both negative and positive area responses, MSOM area response estimates for unobserved species tended toward zero in most instances. This would lead the estimated probability of occurrence of many unobserved species to be comparable among the smallest and largest patches, thus leading to underestimates of the number of species exhibiting preference for large habitat patches and overestimates of the number of species capable of inhabiting small patches.

Overall, observed species counts tended to provide the best estimates of SAR c‐values. However, both iNEXT.3D and MSOMs were able to provide comparable SAR c‐value estimates in certain conditions (Appendix S1: Figure S3). Furthermore, iNEXT.3D and MSOMs almost always provided less biased SAR z‐value estimates than observed counts, which tend to be of greater ecological interest than SAR *c*‐values. For instance, in their assessment of SARs across biogeographical realms, Matthews et al. (2016) only compared SAR *z*‐values, while many empirical habitat fragmentation analyses do not even report *c*‐values (e.g., Litza & Diekmann, 2020; Palmeirim et al., 2021). Based on our results, it thus seems preferable to use MSOMs or iNEXT.3D for estimating species richness and SARs, compared to observed species counts. Ecologists must, however, recognize the potential for inaccuracy in SAR estimation (and particularly underestimated *z*‐values) regardless of the estimator used. Most importantly, our results indicate that researchers should always seek to maximize sampling effort to limit biases in SARs, particularly for larger patches, even when using statistical estimators to correct for imperfect detection.

### 4.2 Beta‐diversity estimates

Observed species counts provided accurate estimates of pairwise Sørensen similarity in almost all simulated scenarios, suggesting that incidence‐based β‐diversity is less sensitive to imperfect detection than species richness. Indeed, previous research suggests β‐diversity estimates can be fairly accurate if the most common species in each assemblage are detected (Cardoso et al., 2009; Roden et al., 2018), though accuracy is also known to improve roughly linearly with sampling completeness (Beck et al., 2013). Our results support this, and we found that observed counts often substantially underestimate Sørensen similarity when mean detection probabilities are low (Appendix S1: Figure S4). Importantly, we show that MSOMs consistently provided improved Sørensen estimate performance over observed counts, and highly accurate Sørensen estimates even when mean detection probabilities were very low.

Furthermore, both observed counts and iNEXT.3D consistently failed to provide accurate estimates of the effects of patch area on β‐diversity (i.e., Sørensen model coefficients), suggesting there were area‐related biases in the associated pairwise Sørensen estimates. Indeed, both observed counts and iNEXT.3D considerably underestimated Sørensen similarity when differences in patch area were low (Appendix S1: Figure S8), leading to the observed underestimates of the intercept, and overestimates of the slope, of β‐diversity models. This suggests that iNEXT.3D provides no advantage over observed counts when investigating covariate effects on β‐diversity. However, MSOMs were able to provide unbiased Sørensen similarity estimates across the full spectrum of differences in patch area, which translated into highly accurate estimates of β‐diversity model coefficients (Appendix S1: Figures S5, S6, S8). We thus recommend that, wherever possible, MSOMs should be used in assessments of covariate effects on pairwise β‐diversity.

### 4.3 Sensitivity to detection probability and sampling design

Unsurprisingly, estimates of all investigated parameters tended to improve with increases in mean species detection probability. Greater variability in detection probabilities across a community also tended to improve estimator performance when mean detection probability was low but had the opposite effect at high mean detection probability. This roughly translates to declines in estimator performance with increases in the proportion of cryptic or rare species, in line with previous research on richness estimators (McNew & Handel, 2015; Poulin, 1998; Tingley et al., 2020). In almost all cases, MSOMs were more robust to variation in the mean and standard deviation of detection probabilities than other estimators.

iNEXT.3D consistently provided the least precise estimates of all parameters apart from SAR intercepts, while the precision of MSOM estimates was almost always comparable to, or better than, observed species counts. Our findings thus support previous work showing that Chao estimators may yield highly variable diversity estimates depending on the observed data, especially when a substantial proportion of species in a community are rare or hard to detect (McNew & Handel, 2015, Poulin, 1998, Tingley et al., 2020), as is common in real‐world communities (Fisher et al., 1943; Novotný & Basset, 2003). Given that ecologists rarely have a priori knowledge of the mean and standard deviation of species detection probabilities within empirical communities, diversity estimators should ideally provide reliable estimates, or at least estimates with a predictable level of error, across the full spectrum of detection probabilities (Iknayan et al., 2014; Kéry & Royle, 2008). Considering that MSOMs consistently provided the most accurate estimates of richness and all β‐diversity parameters and provided estimates of SAR z‐values that were comparable to iNEXT.3D in terms of bias and accuracy, but more precise, our results suggest that MSOMs may be preferable for most empirical applications.

Estimates of SAR coefficients also consistently improved with increases in the number of sampling repetitions, although MSOMs were able to provide accurate estimates of all β‐diversity parameters even when sampling repetitions were low. Nonetheless, given the importance of quantifying both alpha‐ and beta‐diversity accurately (Socolar et al., 2016), our findings suggest that diversity estimators should serve to complement, rather than substitute, a sufficiently appropriate sampling effort (Banks‐Leite et al., 2014). Of course, it would be naïve to ignore the logistical and funding constraints often imposed on empirical field studies, which limit the feasible number of sampling repetitions (Eigenbrod et al., 2011; Pasher et al., 2013), thus limiting the potential to implement MSOMs (Dorazio et al., 2006). However, occupancy models may be applied to spatial, rather than temporal, sampling replicates, lessening associated logistical demands (Dorazio et al., 2011; Noble et al., 2023).

### 4.4 Limitations and further research

Although we used simulation parameter values typical of empirical studies of real landscapes (Taubert et al., 2018) and communities (Matthews et al., 2016), there is the possibility that the evaluated estimators may perform differently when applied to empirical data. For instance, Tingley et al. (2020) found that MSOMs tended to overestimate gamma richness in simulated communities, but underestimated gamma richness in empirical datasets. To conclusively evaluate the influence of imperfect detection on SARs and β‐diversity trends, and the ability of diversity estimators to correct for this, further research should apply estimators to exhaustively sampled real‐world communities. The use of richness estimators in conjunction with SAR models that incorporate the effects of sampling effort (e.g., sampling effort species‐area relationships; Azovsky, 2011, de la Sancha & Boyle, 2019) may also help overcome the biases in SAR coefficient estimates observed here.

We also only analysed the performance of estimators of incidence‐based alpha‐ and beta‐diversity indices in this study. However, much ecological research focuses on patterns of species evenness, which can only be assessed using abundance‐based diversity indices (Barwell et al., 2015). It may thus also be of interest to investigate the relative performance of methods such as iNEXT.3D and Multi‐Species Abundance Models (the abundance‐based equivalent of MSOMs; Mimnagh et al., 2022; Madsen & Royle, 2023), as estimators of trends in abundance‐based alpha‐ and β‐diversity indices. To date, MSAMs have received relatively limited use within fragmentation ecology compared to MSOMs (but see Fogarty et al., 2022). Therefore, to maximize the wider applicability of our findings, we opted to focus our study on MSOMs, and their relative performance compared to observed species counts and iNEXT.3D. Nonetheless, given that MSOMs routinely provided biased estimates of richness and SAR coefficients under our simulation framework, further research may also seek to assess whether MSAMs may perform better than MSOMs as estimators of species richness and SARs, as well as incidence‐based β‐diversity indices and models.

Finally, given the relative paucity of research on the impacts of imperfect detection and covariate‐related biases in β‐diversity estimation, it would be of value to explore estimator performance in different ecological contexts, particularly concerning other drivers of biogeographic variation in species composition, where β‐diversity indices are frequently used (Socolar et al., 2016). A topic of particular interest may be the ability to account for spatial autocorrelation in species identities, and thus community (dis)similarity. We opted not to incorporate spatial autocorrelation in species occurrence within our simulation framework, instead assuming that variation in habitat patch area was the major factor driving variation in community structure among patches. Nonetheless, recent simulation studies suggest that spatial autocorrelation in species occurrence can influence trends in community structure within fragmented landscapes (Ciccheto et al., 2024; Tardanico & Hovestadt, 2023). Furthermore, at larger spatial scales (e.g., across multiple biomes or ecoregions) spatial autocorrelation is likely to be one of, if not the, major contributors to variation in community structure. Given that there are now several spatially explicit estimators of richness and beta‐diversity, including spatial MSOMs (Doser et al., 2022; Johnson et al., 2013), evaluating the combined impacts of spatial autocorrelation and imperfect detection on estimates of β‐diversity trends, and the ability of diversity estimators to correct for these influences, should be a focus of future research (Guelat & Kery, 2018).

## 5 CONCLUSIONS

Although our simulations focused on the area‐related structuring of fragmented communities, our findings reflect widely used sampling approaches and therefore can be generalized to many contexts. Importantly, we show that statistical diversity estimators seldom fully account for biases resulting from imperfect detection and incomplete spatial sampling. The impacts of spatial subsampling have long been recognized as a potential cause of inaccuracies in biodiversity assessments, but in practice, this issue has often been ignored (Azovsky, 2011; Eigenbrod et al., 2011). This study serves to reiterate the need to consider the relative coverage of sampling effort when drawing inference on richness‐covariate relationships (Banks‐Leite et al., 2014). Nonetheless, we show that both iNEXT.3D and MSOMs constitute useful tools for better estimation of the slopes of SARs and other richness models. Furthermore, for the first time, we demonstrate that MSOMs can provide accurate estimates of pairwise β‐diversity model coefficients, even in the most suboptimal scenarios. Nevertheless, given that both alpha‐ and beta‐diversity should be combined to fully characterize biotic assemblages (Socolar et al., 2016), and that richness estimates improved substantially with increased sampling repetitions, regardless of the method used, the value of increased sampling effort cannot be understated.

## AUTHOR CONTRIBUTIONS

**Ciar D. Noble:** Conceptualization (lead); data curation (lead); formal analysis (lead); investigation (lead); methodology (lead); software (lead); visualization (lead); writing – original draft (lead); writing – review and editing (equal). **Carlos A. Peres:** Supervision (equal); writing – review and editing (equal). **James J. Gilroy:** Conceptualization (supporting); methodology (supporting); supervision (equal); validation (lead); writing – review and editing (equal).

## CONFLICT OF INTEREST STATEMENT

The authors declare that they have no competing financial interests or personal relationships that could have influenced the work reported in this paper.

## Supporting information


Appendix S1.

Appendix S2.

## Data Availability

This research was entirely based on simulated data. All code required to replicate the simulated data and associated analyses is available via Zenodo (https://zenodo.org/records/10055523) and a dedicated GitHub repository (https://github.com/ciardorje/Imperfect_Detection_Simulation).
