# Supplementary material for: Accounting for imperfect detection when estimating species‐area relationships and beta‐diversity
Source: Ecol Evol. 2024 Jul 10;14(7):e70017. doi: 10.1002/ece3.70017 (PMC11236461; doi:10.1002/ece3.70017)
Supplement: Supplementary file 1 — Appendix S1. [file ECE3-14-e70017-s002.docx]

**Journal Name:** Ecological Applications

**Title:** Accounting for imperfect detection when estimating species-area relationships and beta diversity

**Authors:** Ciar D. Noble, Carlos A. Peres, James J. Gilroy

**Appendix S1: Supplementary Figures**

- **Table S1:** The area-related sampling design used for all simulated sampling processes.
- **Figure S1:** Performance metrics for estimates of site-level richness under all nine possible detection probability scenarios.
- **Figure S2:** Performance metrics for estimates of pairwise Sørensen similarity under all nine possible detection probability scenarios.
- **Figure S3:** Performance metrics for estimates of Species-Area Relationship z-values under all nine possible detection probability scenarios.
- **Figure S4:** Performance metrics for estimates of Species-Area Relationship c-values under all nine possible detection probability scenarios.
- **Figure S5:** Performance metrics for estimates of the slope of pairwise Sørensen similarity models under all nine possible detection probability scenarios.
- **Figure S6:** Performance metrics for estimates of the intercept of pairwise Sørensen similarity models under all nine possible detection probability scenarios.
- **Figure S7:** The relationship between the bias of species richness estimates and relative patch area.
- **Figure S8:** The relationship between the bias of pairwise Sørensen similarity and the relative pairwise difference in patch area.

**Table S1:** The number of transects placed in each of the 25 patches within each simulated landscape, alongside the total area covered by the *n* transects. We simulated a sampling design where the number of transects placed increased with patch area within each simulated landscape, as is typical in fragmentation ecology. This was done by sorting the patches within each landscape in order of increasing area and then binning into groups of five based on their relative size. Patches within each bin were then assigned the same number of transects, with transect number increasing from the bin containing the smallest five patches to that containing the largest five patches. As patch areas were generated using a stochastic process, the proportion of each patch that was sampled varied among simulation repetitions but was always roughly negatively correlated with patch size within individual simulated landscapes.

| **Patch Area Bin** | **Number of Transects** | **Total Sampled Area (Ha)** |
| --- | --- | --- |
| 1 (Smallest Five Patches) | 1 | 5.93 |
| 2 (2^nd^ Smallest Five Patches) | 2 | 11.86 |
| 3 (Medium Five Patches) | 3 | 17.79 |
| 4 (2^nd^ Largest Five Patches) | 4 | 23.73 |
| 5 (Largest Five Patches) | 5 | 29.66 |


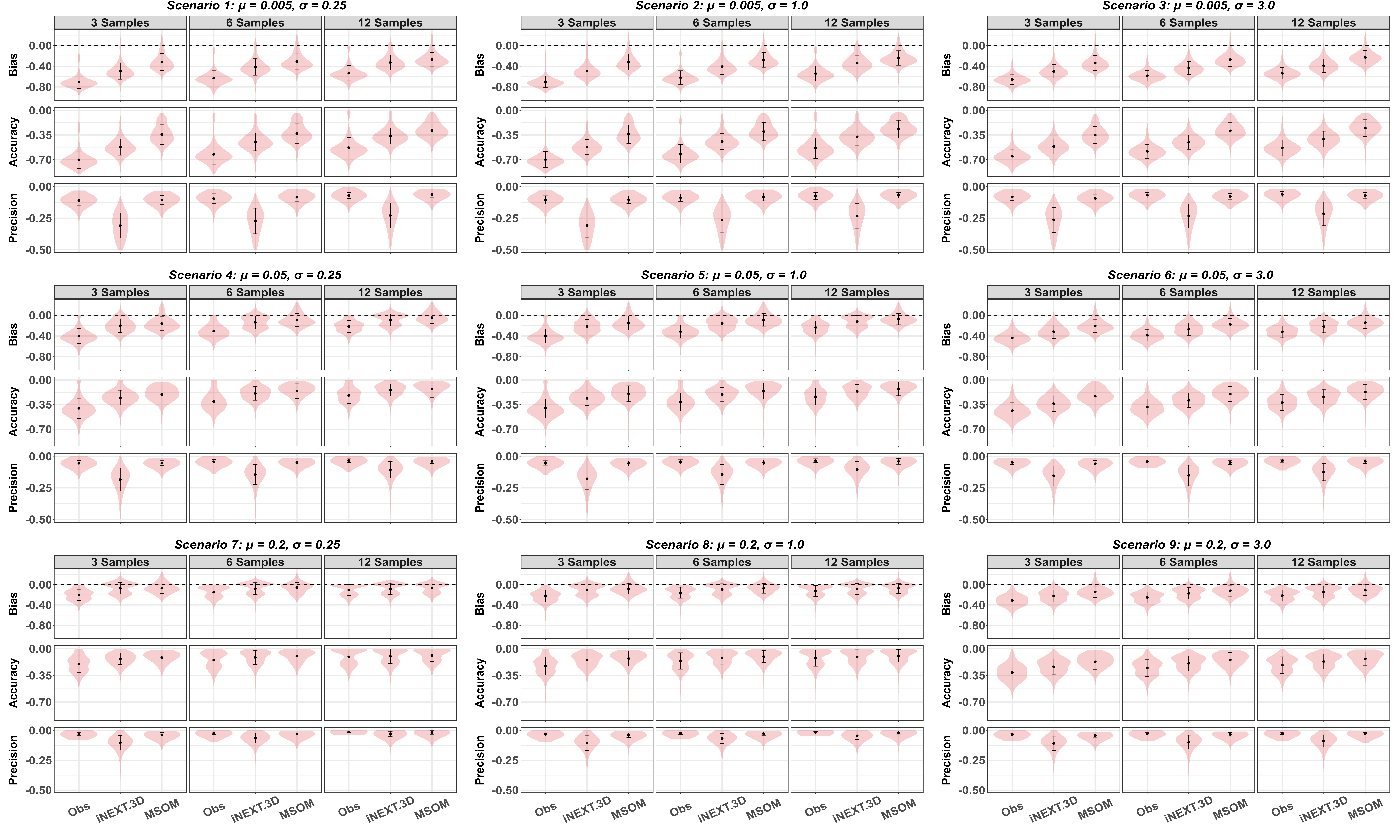


**Figure S1**: Bias, accuracy, and precision of site-level species richness estimates for all 2835 simulated landscape communities (70,875 sites total). Estimates were derived from: observed species counts (Obs), iNEXT.3D and Multi Species Occupancy Models (MSOM). Accuracy and bias are presented in units of percentage difference from the true richness; dashed black lines denote 0 bias values. Results from simulations of all nine detection scenarios are presented in order of increasing mean (µ: top to bottom) and standard deviation (σ: left to right) of the detection probability hyperparameter and are further partitioned by the number of simulated sampling visits to each site. Dots represent the mean, and whiskers the standard deviation, of the metric values.


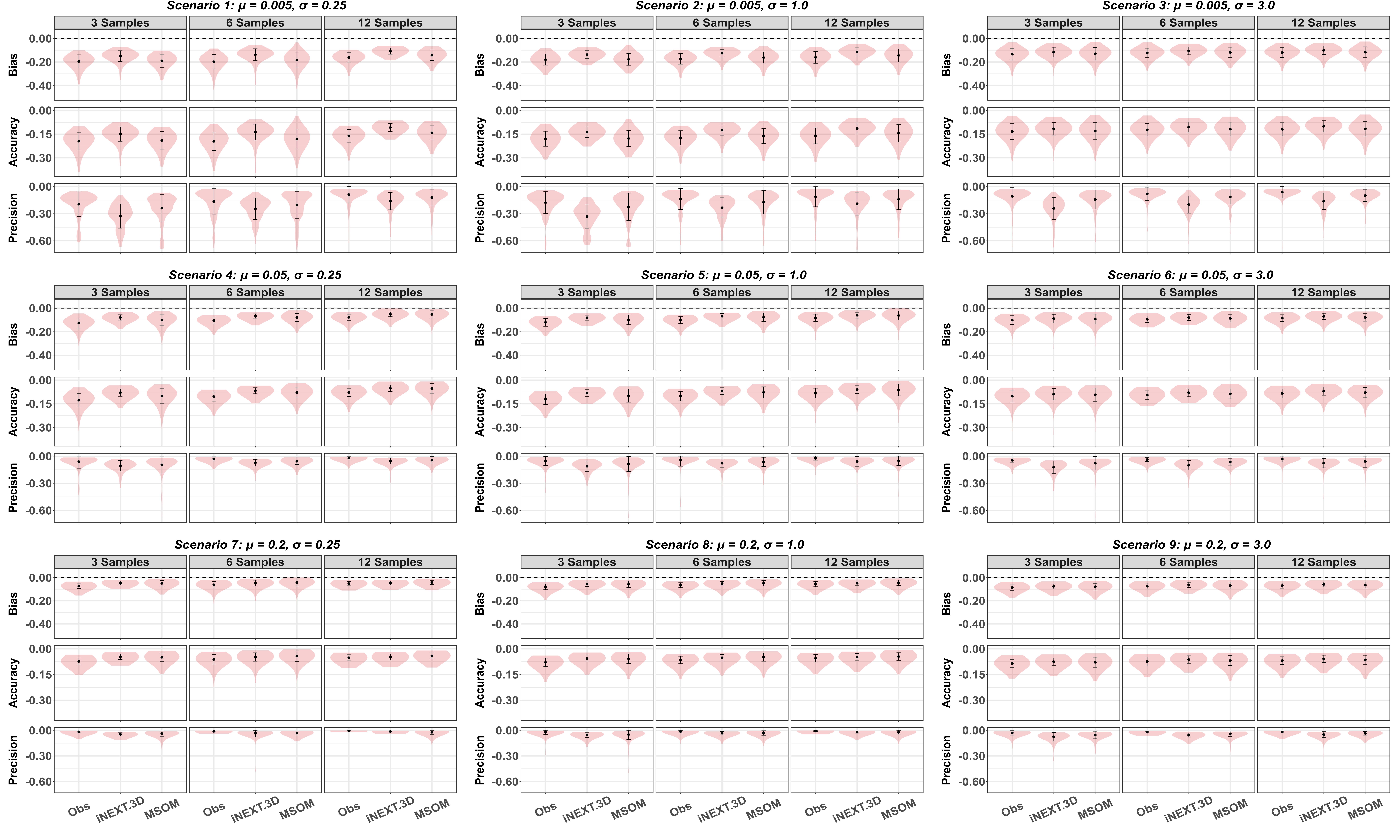


**Figure S2**: Bias, accuracy, and precision of estimates of the *z*-value (slope) of the Species-Area Relationship from all 2835 simulated landscape communities. Estimates were derived from models of site-level richness estimates from: observed species counts (Obs), iNEXT.3D and Multi Species Occupancy Models (MSOM). Dashed black lines denote 0 bias values. Results from simulations using all nine possible detection scenarios are presented in order of increasing mean (µ: top to bottom) and standard deviation (σ: left to right) of the detection probability hyperparameter and are further partitioned by the number of simulated sampling visits to each site. Dots represent the mean, and whiskers the standard deviation, of the metric values.


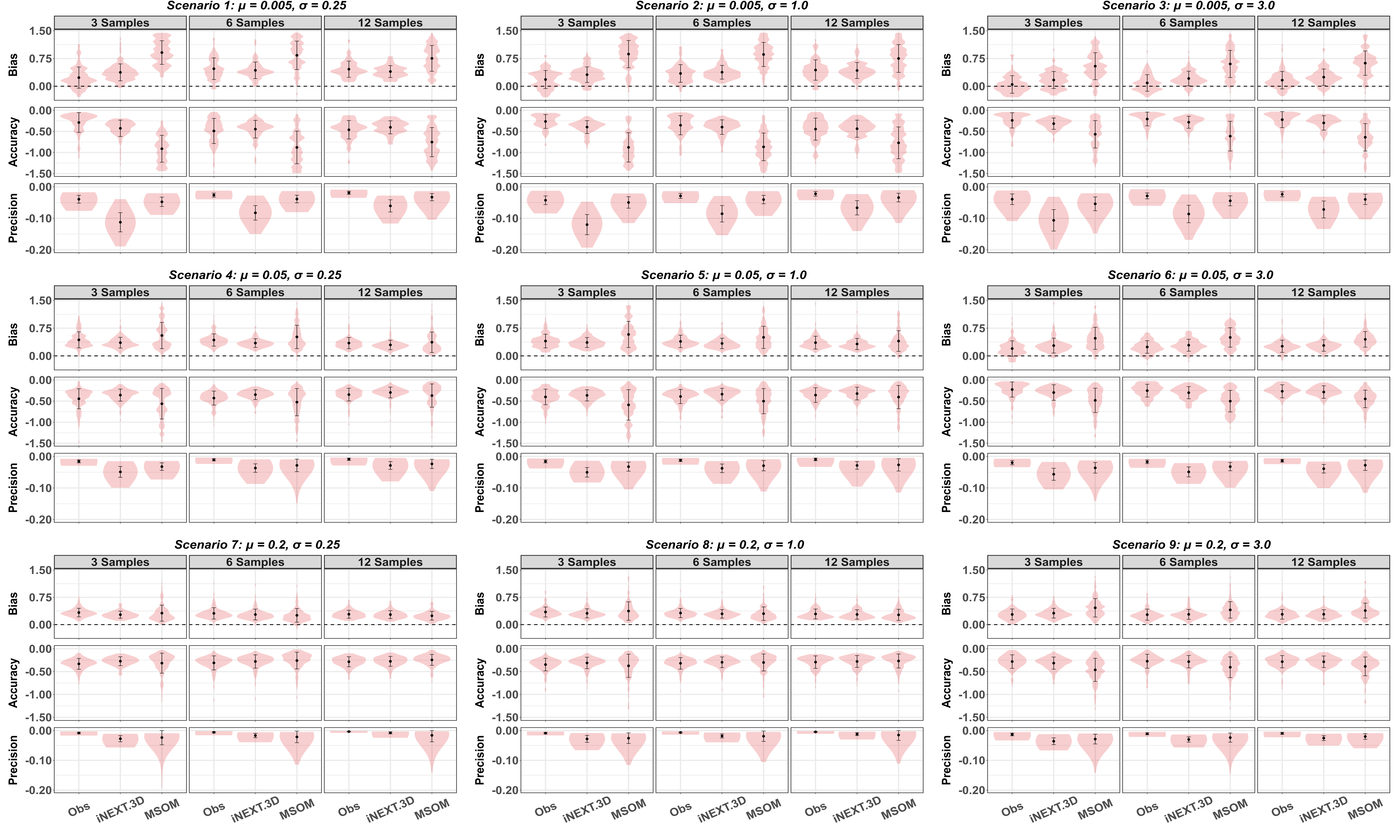


**Figure S3**: Bias, accuracy, and precision of estimates of the *c*-value (intercept) of the Species-Area Relationship from all 2835 simulated landscape communities. Estimates were derived from models of site-level richness estimates from: observed species counts (Obs), iNEXT.3D and Multi Species Occupancy Models (MSOM). Dashed black lines denote 0 bias values (i.e., no difference between true and estimated richness). Results from simulations using all nine possible detection scenarios are presented in order of increasing mean (µ: top to bottom) and standard deviation (σ: left to right) of the detection probability hyperparameter. Results are further partitioned by the number of simulated sampling visits to each site. Dots represent the mean, and whiskers the standard deviation, of the metric values.


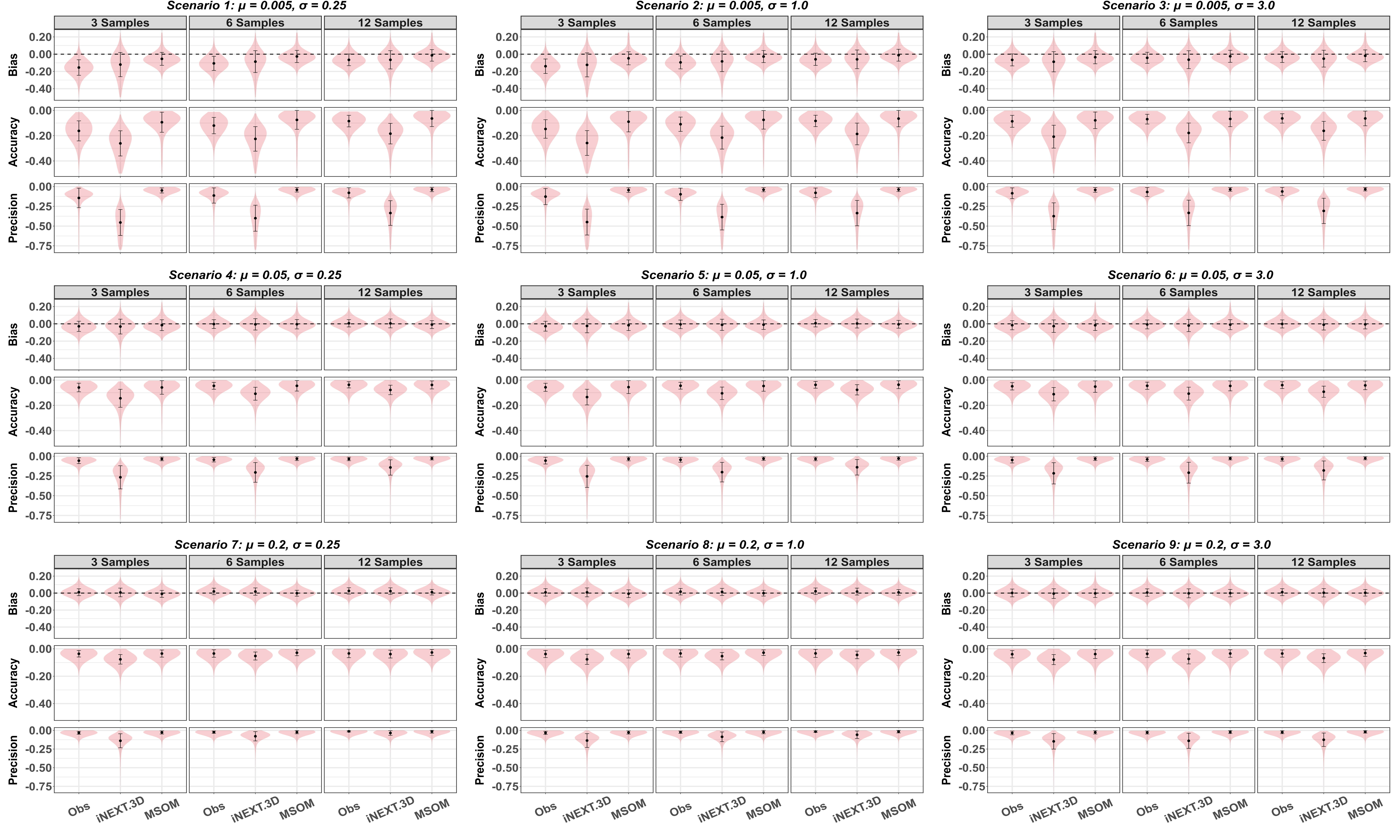


**Figure S4**: Bias, accuracy, and precision of pairwise Sørensen similarity estimates from all 2835 simulated landscape communities (850,500 site pairs total). Estimates were derived from: observed species counts (Obs), iNEXT.3D and Multi Species Occupancy Models (MSOM). Dashed black lines denote 0 bias values. Results from simulations using all nine possible detection scenarios are presented in order of increasing mean (µ: top to bottom) and standard deviation (σ: left to right) of the detection probability hyperparameter and are further partitioned based on the number of simulated sampling visits to each site. Dots represent the mean, and whiskers the standard deviation, of the metric values.


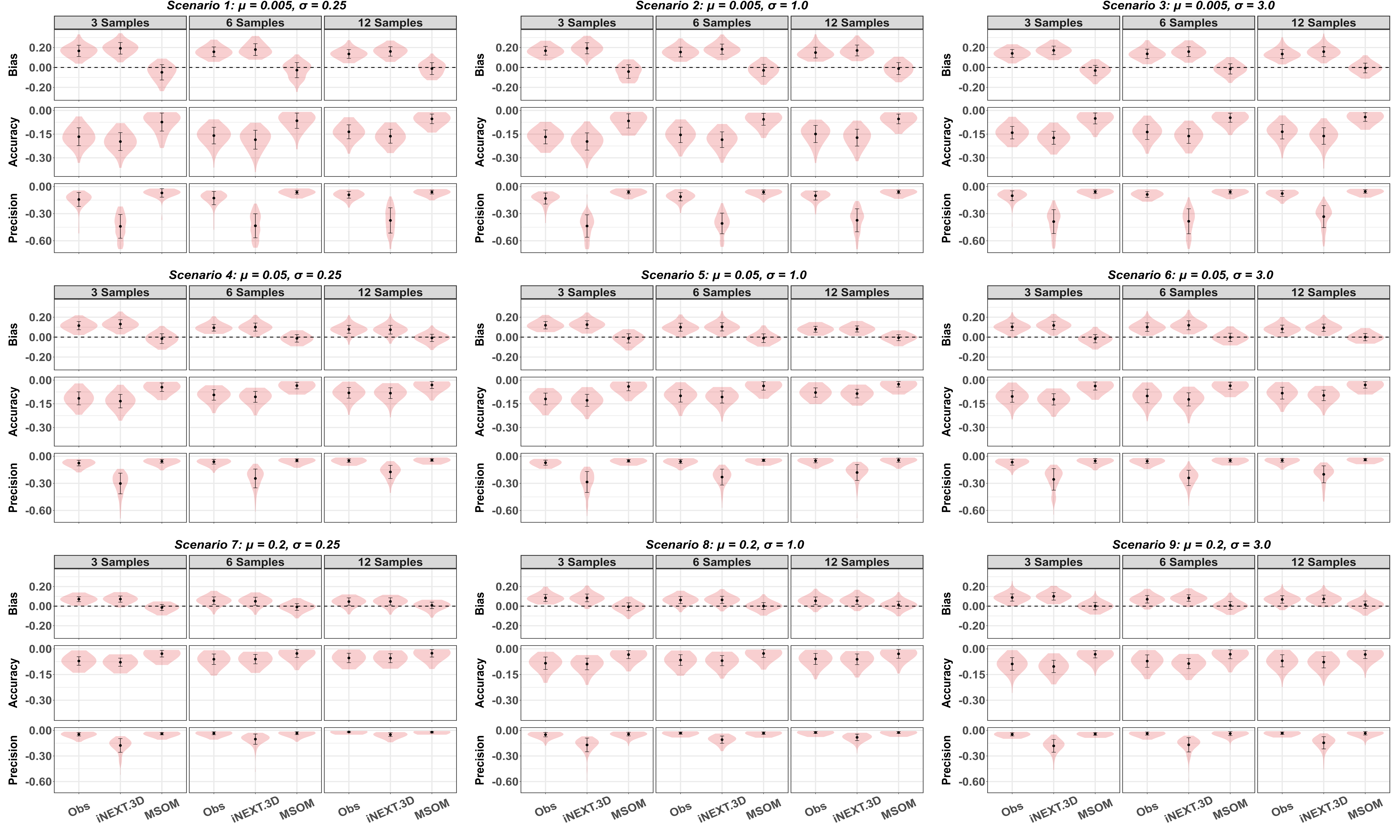


**Figure S5**: Bias, accuracy, and precision of estimates of the slope of pairwise Sørensen similarity models from all 2835 simulated landscape communities. Estimates were derived from models of pairwise Sørensen similarity estimates from: observed species counts (Obs), iNEXT.3D and Multi Species Occupancy Models (MSOM). Dashed black lines denote 0 bias values (i.e., no difference between true and estimated richness). Results from simulations using all nine possible detection scenarios are presented in order of increasing mean (µ: top to bottom) and standard deviation (σ: left to right) of the detection probability hyperparameter. Results are further partitioned by the number of simulated sampling visits to each site. Dots represent the mean, and whiskers the standard deviation, of the metric values.


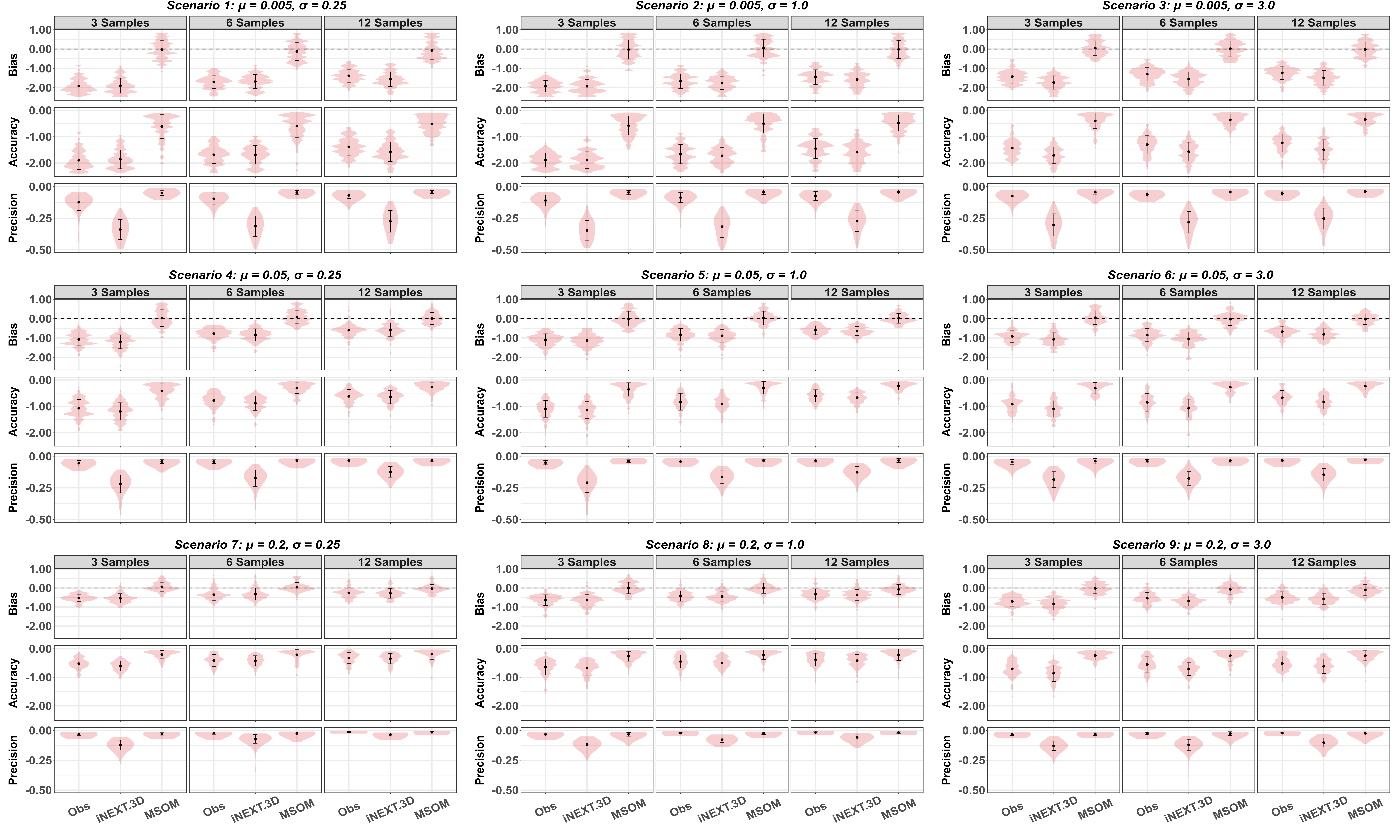


**Figure S6**: Bias, accuracy, and precision of estimates of the intercept of pairwise Sørensen similarity models from all 2835 simulated landscape communities. Estimates were derived from models of pairwise Sørensen similarity estimates from: observed species counts (Obs), iNEXT.3D and Multi Species Occupancy Models (MSOM). Dashed black lines denote 0 bias values (i.e., no difference between true and estimated richness). Results from simulations using all nine possible detection scenarios are presented in order of increasing mean (µ: top to bottom) and standard deviation (σ: left to right) of the detection probability hyperparameter. Results are further partitioned by the number of simulated sampling visits to each site. Dots represent the mean, and whiskers the standard deviation, of the metric values.


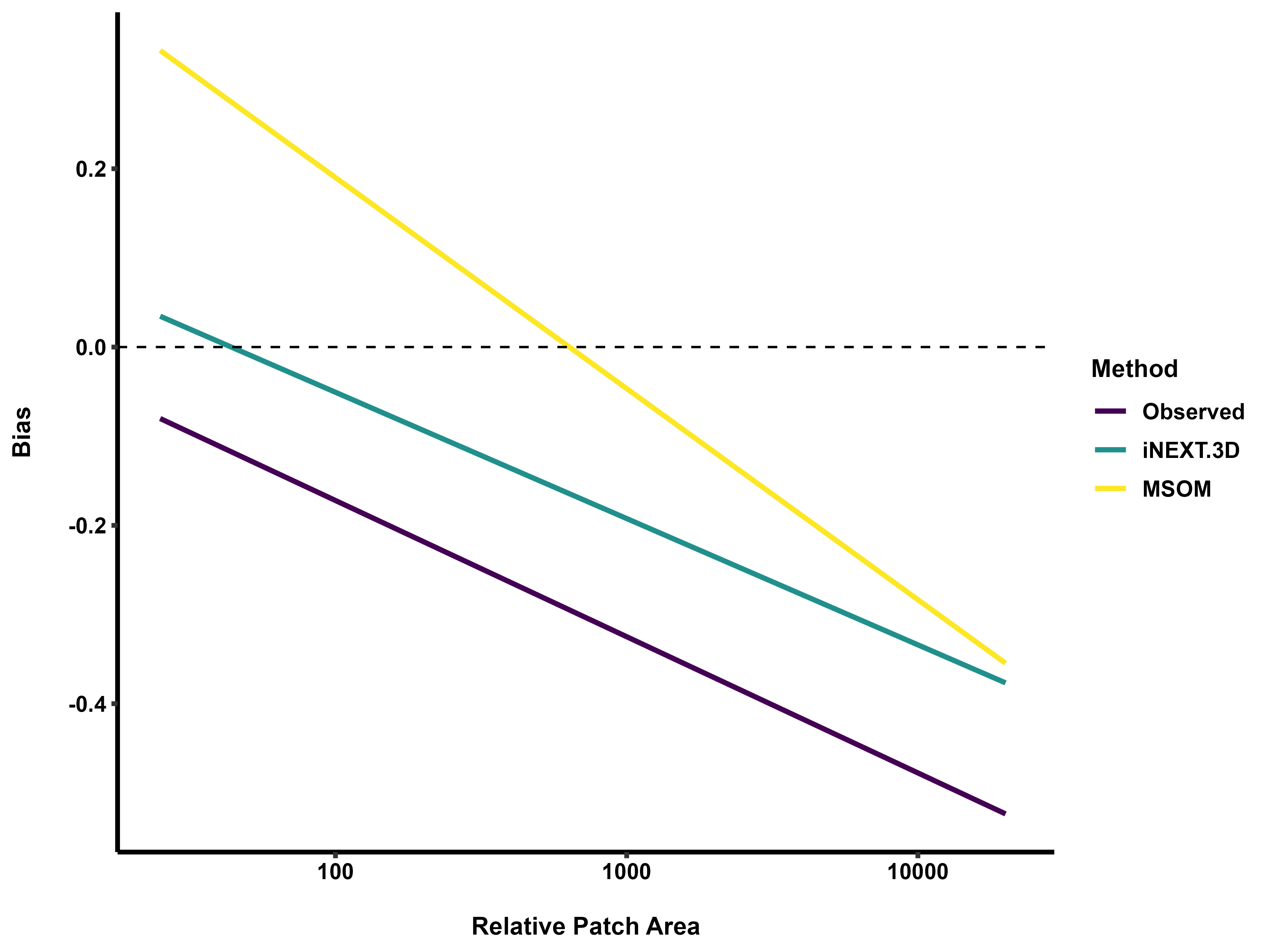


**Observed: β = -0.153 iNEXT.3D: β = -0.142 MSOM: β = -0.237**

**Figure S7**: The relationship between relative patch area and the bias (in units of % difference from true richness) of site-level species richness estimates derived from observed species counts (Observed), iNEXT.3D and Multi Species Occupancy Models (MSOM), across all 2835 simulated landscapes (70,875 sites total). Coloured lines represent the slope of the fixed effect of log_10_-transformed patch area on estimate bias (β). The dashed black line denotes 0 bias (i.e., no difference between the true and estimated richness). Slopes were estimated using multiple linear regression, to avoid confounding the effect of patch area. Models also included fixed effects of 1) the mean and 2) the standard deviation of the detection probability hyperparameter used, and 3) the number of simulated repeat sampling visits. The effect of patch area on estimate bias was significant in all cases (p < 0.001).

**Figure S8**: The relationship between the relative pairwise difference in patch area and the bias of pairwise Sørensen similarity estimates derived from observed species counts (Observed), iNEXT.3D and Multi Species Occupancy Models (MSOM), across all 2835 simulated landscapes (850,500 site pairs total). Coloured lines represent the slope of the fixed effect of log_10_-transformed difference in patch area on estimate bias (β). The dashed black line denotes 0 bias (i.e., no difference between the true and estimated Sorensen similarity). Slopes were estimated using multiple linear regression, to avoid confounding the effect of patch area. Models also included fixed effects of 1) the mean and 2) the standard deviation of the detection probability hyperparameter used, and 3) the number of simulated repeat sampling visits. The effect of difference in patch area on estimate bias was significant in all cases (p < 0.001).


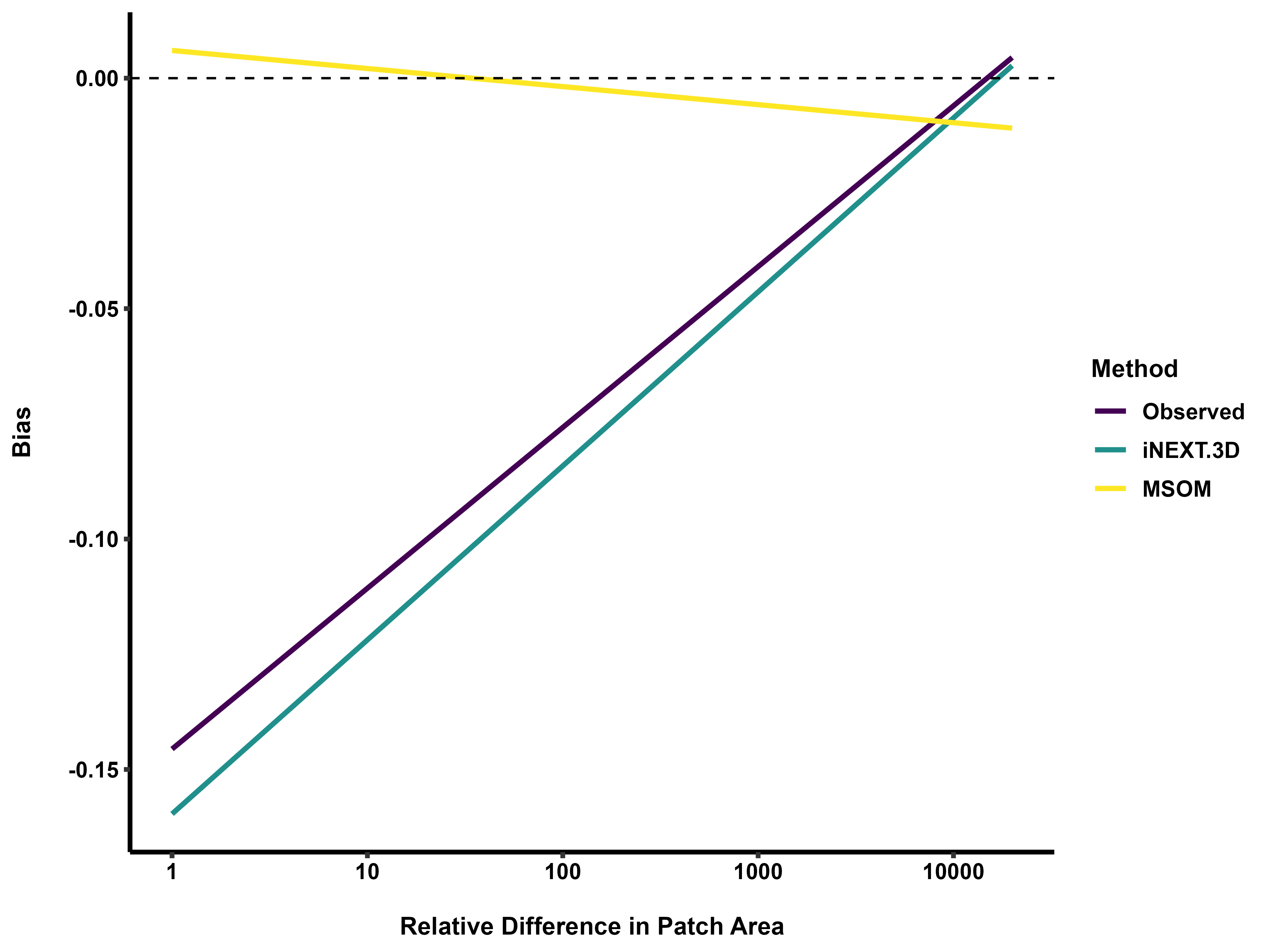


**Observed: β = 0.035 iNEXT.3D: β = 0.038 MSOM: β = -0.004**
