# Supplementary material for: Accounting for imperfect detection when estimating species‐area relationships and beta‐diversity
Source: Ecol Evol. 2024 Jul 10;14(7):e70017. doi: 10.1002/ece3.70017 (PMC11236461; doi:10.1002/ece3.70017)
Supplement: Supplementary file 2 — Appendix S2. [file ECE3-14-e70017-s001.pdf]

Appendix S2: NIMBLE Code for Multi-Species Occupancy Model

Equation S1: Bayesian Hierarchical Multi-Species Occupancy Model used to estimate patch-level richness and pairwise Sørensen similarity

```
HMSOM <- nimbleCode({

  #Community-Level Hyperparameters#
  #Community inclusion parameter - approximation of Link's scale prior
  omega ~ dbeta(0.001, 1)

  psi.mean ~ dbeta(1, 1) #Approximate Uniform
  mu.lpsi <- logit(psi.mean)
  sd.lpsi ~ dunif(0, 5)
  tau.lpsi <- 1/sd.lpsi^2
  p.mean ~ dbeta(1, 1) #Approximate Uniform
  mu.lp <- logit(p.mean)
  sd.lp ~ dunif(0, 5)
  tau.lp <- 1/sd.lp^2

  mu.Area ~ dnorm(0,0.1)
  sd.Area ~ dunif(0, 5)
  tau.Area <- 1/sd.Area^2

  #Correlation between occurrence and detection probabilities
  rho ~ dunif(-1, 1)
  tau.eta <- tau.lp/(1 - rho^2)

  for(k in 1:M){

    #Species-Level Priors#
    w[k] ~ dbern(omega)
    lpsi[k] ~ dnorm(mu.lpsi, tau.lpsi)
    bArea[k] ~ dnorm(mu.Area, tau.Area)
    mu.eta[k] <- mu.lp + rho * sd.lp/sd.lpsi * (lpsi[k] - mu.lpsi)
    lp[k] ~ dnorm(mu.eta[k], tau.eta)

    #Likelihood#
    #Detection probability - consistent among patches (i.e., intercept only)
    logit(p[k]) <- lp[k]

    for(i in 1:nPatches){

      #Non-conditional occurrence probability
      logit(psi[i, k]) <- lpsi[k] + bArea[k] * Area[i]
      #Binary occurrence indicator
      z[i, k] ~ dbern(psi[i, k] * w[k])
      #Model observation records
      y[i, k] ~ dbin(p[k] * z[i, k], nSamples)

    }
  }
})
```
